# Reduction of ventilatory time using the multidisciplinary disconnection protocol. Pilot study*

**DOI:** 10.1590/1518-8345.2923.3215

**Published:** 2019-12-05

**Authors:** Miriam Sánchez-Maciá, Jaime Miralles-Sancho, María José Castaño-Picó, Ana Pérez-Carbonell, Loreto Maciá-Soler

**Affiliations:** 1 Hospital General Universitario de Elche, Elche, Spain.; 2 Universidad de Alicante, Alicante, Spain.

**Keywords:** Respiration Artificial, Nursing Assessment, General Surgery, Critical Care, Evidence-Based Practice, Postoperative Period, Respiração Artificial, Avaliação em Enfermagem, Cirurgia Geral, Cuidado Crítico, Prática Clínica Baseada em Evidências, Período Pós-Operatório, Respiración Artificial, Evaluación en Enfermería, Cirugía General, Cuidados Críticos, Práctica Clínica Basada en la Evidencia, Periodo Posoperatorio

## Abstract

**Objective:**

compare ventilatory time between patients with the application of a disconnection protocol, managed in a coordinated way between doctor and nurse, with patients managed exclusively by the doctor.

**Method:**

experimental pilot study before and after. Twenty-five patients requiring invasive mechanical ventilation for 24 hours or more were included, and the protocol-guided group was compared with the protocol-free group managed according to usual practice.

**Results:**

by means of the multidisciplinary protocol, the time of invasive mechanical ventilation was reduced (141.94 ± 114.50 vs 113.18 ± 55.14; overall decrease of almost 29 hours), the time spent on weaning (24 hours vs 7.40 hours) and the numbers of reintubation (13% vs 0%) in comparison with the group in which the nurse did not participate. The time to weaning was shorter in the retrospective cohort (2 days vs. 5 days), as was the hospital stay (7 days vs. 9 days).

**Conclusion:**

the use of a multidisciplinary protocol reduces the duration of weaning, the total time of invasive mechanical ventilation and reintubations. The more active role of the nurse is a fundamental tool to obtain better results.

## Abstract (pt)

**Objetivo:**

comparar o tempo ventilatório em pacientes submetidos a desconexão segundo um protocolo conduzido de forma coordenada pelo médico e pela enfermeira com o mesmo tempo em pacientes tratados exclusivamente pelo médico.

**Método:**

estudo piloto experimental antes e depois. Foram incluídos 25 pacientes que precisaram de ventilação mecânica invasiva durante 24 horas ou mais, e o grupo orientado por protocolo foi comparado com o grupo sem protocolo, tratado conforme a prática habitual.

**Resultados:**

mediante o protocolo multidisciplinar, conseguiu-se reduzir o tempo de ventilação mecânica invasiva (141,94 ± 114,50 vs. 113,18 ± 55,14; redução global de quase 29 horas), o tempo empregado no desmame (24 horas vs. 7,40 horas) e as cifras de reintubação (13% vs. 0%) em comparação com o grupo em que não houve participação da enfermeira. O tempo até iniciar o desmame foi menor na coorte retrospectiva (2 dias vs. 5 dias), bem como a internação hospitalar (7 dias vs. 9 dias).

**Conclusão:**

a adoção de um protocolo multidisciplinar diminui a duração do desmame, o tempo total de ventilação mecânica invasiva e as reintubações. O papel mais ativo da enfermeira é considerado uma ferramenta fundamental para obter melhorias nos resultados.

## Abstract (es)

**Objetivo:**

comparar el tiempo ventilatorio entre pacientes sometidos a desconexión según un protocolo manejado de forma coordinada por el médico y la enfermera con el mismo tiempo en pacientes manejados exclusivamente por el médico.

**Método:**

estudio piloto experimental antes y después. Se incluyeron a 25 pacientes que requirieron ventilación mecánica invasiva durante 24 horas o más, y se comparó el grupo orientado por protocolo con el grupo sin protocolo, manejado según la práctica habitual.

**Resultados:**

mediante el protocolo multidisciplinar se logró disminuir el tiempo de ventilación mecánica invasiva (141,94 ± 114,50 vs. 113,18 ± 55,14; disminución global de casi 29 horas), el tiempo empleado en el destete (24 horas vs. 7,40 horas) y las cifras de reintubación (13 % vs. 0%) en comparación con el grupo en el que no participó la enfermera. El tiempo hasta iniciar el destete fue menor en la cohorte retrospectiva (2 días vs. 5 días), así como también la estancia hospitalaria (7 días vs. 9 días).

**Conclusión:**

la adopción de un protocolo multidisciplinar disminuye la duración del destete, el tiempo total de ventilación mecánica invasiva y las reintubaciones. El papel más activo de la enfermera se considera una herramienta fundamental para obtener mejoras en los resultados.

## Introduction

Mechanical ventilation (MV) is one of the most commonly techniques used in Intensive Care Units, and its disconnection is one of the most evaluated procedures based on scientific evidence^(^ 1 ^-^ 5 ^)^. Currently, the invasive mechanical ventilation (IMV) removal process occupies about 40%^(^ 1 ^,^ 6 ^-^ 8 ^)^ of the total ventilatory support time, representing a great difficulty for both the patient and the professional. The more difficult are to remove ventilatory support, the greater the chances of suffering complications such as airway trauma or nosocomial infection, among others, which in turn would lead to an increase in hospital stay, costs or mortality, also having repercussions on the patient’s quality of life^(^ 6 ^)^, reasons to try to shorten ventilatory time.

The use of disconnection protocols brings efficacy to daily clinical practice and avoids individual judgement based on one’s own experience, reducing variability in the disconnection process^(^ 4 ^,^ 9 ^)^. It is possible to reduce the total duration of mechanical ventilation in 26% and the stay in the Critical Care Unit in 11% without repercussions in patient’s morbidity and mortality^(^ 9 ^)^ with the application of release protocols, considering how important is the role of the nurse within the process, contributing to improvements in the reduction of the stay in hospital^(^ 3 ^,^ 10 ^)^.

However, despite the published data, the disconnection of IMV remains a process with a lack of consensus^(^ 9 ^)^, and this is why the research in this field is justified.

Our main objective was to compare ventilatory time between patients with the application of a disconnection protocol managed in a coordinated way between doctor and nurse versus patients managed exclusively by the doctor. Our secondary objectives were to compare the rate of reintubation between the two cohorts, to compare the duration of weaning, and to compare the days of stay in the unit between the two groups of patients.

## Method

An experimental before and after pilot study was carried out in the Resuscitation Unit of the *Hospital General Universitario de Elche*, which consists of six critical care beds for surgical patients. This pilot study was performed to verify if the mechanical ventilation disconnection protocol managed in a multidisciplinary way was effective and with the intention to continue later a multicenter study of cases and control, if the results were favorable. The ethics committee of the *Hospital General Universitario de Elche* approved the work, and informed consents were obtained from the relatives of the patients who were included in the prospective group.

Before starting the study, two half-hour meetings were held to explain the study, the protocol, how to carry it out and how to complete the data collection notebook. In addition, the research team was available to answer questions from both the medical team and the nursing team. The data collection notebook was the only instrument used for the collection of information.

All patients over the age of eighteen who were admitted in the Resuscitation unit, who required IMV for a period greater than or equal to 24 hours, who were extubated, and who had either signed the informed consent form to participate in the study, or their relatives, were included. All patients who died during the period of MV and those who ended up tracheostomized after a period of MV were excluded.

**Figure 1:**
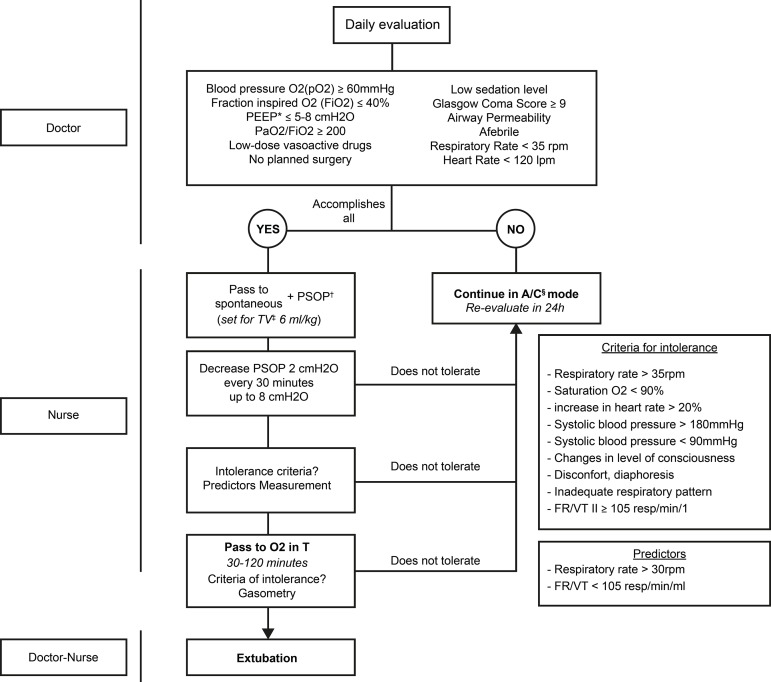
Weaning Algorithm *PEE = Tele-Expiratory Pressure; †PSOP = Pressure Support; ‡TV = Tidal Volume; §A/C = Assisted/Controlled; ||FR/VT = Rapid Superficial Respiration Index

Source: Modified from Sánchez-Maciá M, Castaño-Picó MJ, Antón-Latour MA, Maciá-Soler L. Design and implantation of an invasive mechanical ventilation weaning protocol in postoperative patients. Rev ROL Enferm. 2018 Jan; 41(1):28-36.

Twenty-five patients participated in the study. Retrospective data were obtained by reviewing the medical records of patients admitted in the unit during 2014 and who met the inclusion and exclusion criteria. This group had been extubated according to standard clinical practice and at the criteria of the doctor responsible for the patient at that time. Prospective data consisted to all patients who met the inclusion and exclusion criteria during the period between 1 May 2015 and 1 August 2015. In this group, the protocol of disconnection of mechanical ventilation managed in a multidisciplinary way between doctors and nurses was used. The algorithm is shown in Figure 1 and explained below:

1. The doctor was responsible for checking daily^(^ 1 ^-^ 3 ^,^ 5 ^,^ 11 ^-^ 16 ^)^ if the reason for IMV instauration was solved or if there was any improvement^(^ 2 ^,^ 5 ^,^ 7 ^-^ 8 ^,^ 11 ^-^ 12 ^,^ 14 ^,^ 17 ^-^ 21 ^)^ by clinical evaluation, chest x-ray, arterial blood gasometry or any necessary diagnostic test. In addition, a series of criteria for disconnection^(^ 1 ^,^ 5 ^,^ 9 ^,^ 17 ^)^ of MV were evaluated, which the patient had to accomplish completely in order to progress within the protocol:
  a) Respiratory stability: Blood pressure of oxygen (PO2) ≥ 60 mmHg^5^^,^ 7 ^,^ 14 ^,^ 18 ^,^ 22 with inspired oxygen fraction (FiO2) ≤ 0.4 ^(^ 5 ^,^ 10 ^,^ 14 ^,^ 16 ^,^ 19 ^,^ 21 ^-^ 22 ^)^; respiratory rate (RR) < 35 respirations per minute ^(^ 5 ^,^ 19 ^,^ 22 ^-^ 23 ^)^ and positive end-expiratory pressure level (PEEP) ≤ 5-8 cm H2O ^(^ 2 ^,^ 5 ^,^ 7 ^,^ 9 ^-^ 11 ^,^ 13 ^-^ 14 ^,^ 19 ^,^ 21 ^,^ 24 ^-^ 26 ^)^.
  b) Hemodynamic stability with stable cardiovascular function ^(^ 5 ^,^ 14 ^,^ 16 ^,^ 22 ^-^ 23 ^)^, heart rate (HR) < 120 beats per minute^(^ 5 ^,^ 7 ^-^ 8 ^)^ and no need for vasoactive drugs or a minimum amount^(^ 2 ^,^ 5 ^,^ 7 ^-^ 8 ^,^ 11 ^,^ 13 ^,^ 18 ^,^ 20 ^-^ 23 ^)^, accepting doses of less than 5µ/kg/min of dobutamine^(^ 2 ^,^ 5 ^,^ 7 ^,^ 19 ^,^ 22 ^,^ 25 ^)^ and <0.1µg/kg/min of noradrenaline.
  c) Neurological Stability: Glasgow Coma Scale (GCS) ≥ 9^(^ 5 ^,^ 18 ^)^ and between -2 and 0^(^ 5 ^,^ 18 ^)^ on the Richmond Scale^(^ 27 ^)^ to ensure a low level of sedation ^(^ 2 ^,^ 5 ^,^ 11 ^,^ 15 ^,^ 19 ^)^.
  d) Absence of fever^(^ 5 ^,^ 7 ^,^ 16 ^,^ 20 ^,^ 22 ^)^ or hypothermia^(^ 5 ^,^ 16 ^)^, presence of cough reflex ^(^ 2 ^,^ 5 ^,^ 8 ^,^ 13 ^-^ 16 ^,^ 21 ^-^ 22 ^,^ 28 ^)^ and permeable airway ^(^ 2 ^,^ 5 ^,^ 7 ^)^.
2. If the patient complied with all criteria, the nurse connected the patient in a spontaneous mode with support pressure (SP)^(^ 2 ^,^ 5 ^,^ 11 ^-^ 12 ^,^ 16 ^,^ 19 ^,^ 23 ^,^ 29 ^)^, assuring a tidal volume (TV) of 6-8 ml/kg^(^ 5 ^,^ 14 ^,^ 16 ^,^ 25 ^,^ 29 ^-^ 30 ^)^ of ideal body weight, making pressure decreases^(^ 5 ^,^ 7 ^,^ 9 ^,^ 18 ^-^ 19 ^,^ 31 ^)^ of 2 in 2 cm of H2O every 20-30 min^(^ 5 ^)^ until achieving a SP less than or equal to 8 cm H2O^(^ 1 ^-^ 2 ^,^ 5 ^,^ 7 ^,^ 29 ^,^ 32 ^)^. After each decrease, the patient’s tolerance was checked by measuring parameters such as: HR ^(^ 5 ^,^ 8 ^,^ 13 ^,^ 15 ^,^ 18 ^,^ 21 ^-^ 22 ^)^, systolic blood pressure^(^ 5 ^,^ 9 ^,^ 17 ^,^ 26 ^,^ 30 ^)^, O2 saturation^(^ 5 ^,^ 8 ^,^ 13 ^,^ 15 ^,^ 18 ^,^ 21 ^-^ 23 ^)^, level of consciousness^(^ 5 ^,^ 16 ^,^ 20 ^,^ 28 ^)^, presence of discomfort or diaphoresis^(^ 5 ^,^ 8 ^,^ 13 ^,^ 15 ^,^ 18 ^,^ 21 ^-^ 23 ^)^ or tachypnea ^(^ 5 ^,^ 8 ^,^ 13 ^,^ 15 ^,^ 18 ^,^ 21 ^-^ 23 ^,^ 25 ^)^.
3. If the patient tolerated all the changes made, the nurse suspended the IMV with respirator and passed the T-piece oxygen test for 30-120 minutes^(^ 2 ^,^ 5 ^,^ 7 ^,^ 12 ^,^ 14 ^-^ 15 ^,^ 22 ^,^ 32 ^)^, remeasuring the same intolerance criteria as in the previous phase and, in addition, the predictors respiratory rate <35rpm and rapid superficial respiration rate (Respiratory Rate/Tidal Volume) < 105 resp/min/^(^ 1 ^,^ 5 ^,^ 7 ^-^ 8 ^,^ 11 ^-^ 12 ^,^ 14 ^-^ 15 ^,^ 18 ^-^ 19 ^,^ 22 ^-^ 23 ^)^.
4. If the patient was still stable, the extraction of arterial gasometry was carried out, and doctor and nurse carried out extubation jointly if they did not find any alteration. Successful weaning was considered when the patient was able to remain breathing without invasive support for a period greater than or equal to 48 hours^(^ 5 ^,^ 7 ^-^ 9 ^,^ 11 ^-^ 12 ^,^ 22 ^-^ 23 ^,^ 25 ^)^.
5. If the patient did not tolerate the changes made at any point of the protocol or was not a candidate for weaning because the established criteria were not met, mechanical ventilation was resumed in Assisted-Controlled (A/C) mode^(^ 5 ^,^ 7 ^-^ 9 ^)^ and weaning was not attempted again until the following day^(^ 5 ^,^ 7 ^,^ 13 ^-^ 14 ^,^ 18 ^,^ 22 ^,^ 25 ^)^.

The variables studied were, as sociodemographic variables, age and sex; as a result variable, mechanical ventilation time; as explanatory variables, presence of comorbidities measured; as Charlson comorbidity index value^(^ 33 ^)^, time spent in the unit, time spent in weaning, reintubations, time until the weaning process begins since the admission of the patient, classification of anesthetic risk ASA (American Society of Anesthesiologists) and the classifier Acute Physiology and Chronic Health Evaluation. (APACHE) II.

Statistical Package for the Social Sciences (SPSS) 21.0 was used for data analysis. Dichotomous qualitative variables such as sex and reintubations were expressed as percentages, and for comparison, contingency tables and the Fisher test were used. Continuous quantitative variables such as mechanical ventilation time, unit stay, weaning time and time to weaning start were expressed as average ± standard deviation (SD) and/or median (Q1-Q3) if the distribution was normal or not and compared according to the Mann-Whitney test. Correlation tests were also performed between the mechanical ventilation time variable and the rest of the study variables, using the Spearman test for the comparison between two numerical variables. The Mann-Whitney U test was applied for the comparison of a quantitative variable with a qualitative variable when the qualitative variable had two levels and the Kruskall-Wallis test when it was represented in three or more levels. A p value of less than 0.05 was used as statistical significance.

## Results

Twenty-five patients were included in the study, nine in the prospective group and sixteen in the retrospective group. In the retrospective data, nine patients who could not enter in the weaning phase because they were underwent a tracheostomy and one patient due to death were discarded, while in the prospective data there was no loss. The variables studied and their comparison are shown in Table 1.

**Table 1:** Comparison of the variables studied. Elche, CV, Spain, 2015

Variables	Retrospective data (n=16)	Prospective data (n=9)	P value
Male (%)	56.25	67	0.524
Age in years (x̅)	74(q1-q3:65-79)	75(q1-q3:69-81)	0.678
Type of surgery (%):			
General Surgery	50	78	1
Neurosurgery	32	0	
Urology	6.2	11	
Vascular Surgery	6.2	11	
Traumatology	6.2	0	
CCI*(x̅)	5.54±2.31	6.12±2.50	0.635
ASA^†^ (x̅)	3(q1-q3:2.00-3.25)	4(q1-q3:3-4)	0.564
Staying in days (x̅)	7(q1-q3:5.25-10.75)	9(q1-q3:5-10.50)	0.014
Weaning in hours (x̅)	24(q1-q3:24-48)	7.40(q1-q3:3-17.70)	0.004
Time until weaning starts from input (x̅)	2(q1-q3:1-3)	5.00(q1-q3:2-7)	0.122
Total time of VMI^‡^ (x̅)	141.94±114.50	113.18±55.14	0.011
Reintubations: Yes (%)	13	0	0.004

* CCI = Charlson Comorbidity Index;

† ASA = American Society of Anesthesiologists;

‡ IMV = Invasive mechanical ventilation

Referring to sociodemographic characteristics, both groups were comparable in terms of sex (p=0.524), age (p=0.678), ASA classification (p=0.564), comorbidity measured by the Charlson Comorbidity Index^(^ 33 ^)^ (p=0.635) and the type of surgery (p=1.00), finding in the two samples a predominant percentage of patients operated in general surgery (50% vs 78%). It is important to note that both groups had a high comorbidity index (94% vs 89%), which also correlates with a high ASA classification (18% vs 45%), and an age above 70 years.

As for the characteristics most closely related to IMV, the most significant difference between the two groups was found in weaning duration time (p=0.004), hospital stay time (p=0.014) and total IMV time (p=0.011). In mechanical ventilation time, an important reduction was observed in the group in which the multidisciplinary protocol was used as compared to the extubated group according to individual criteria (141.94±114.50 vs 113.18±55.14), achieving an overall decrease in the total mechanical ventilation time of almost 29 hours. There was also an important decrease in the hours used to weaning the patient with the use of the multidisciplinary protocol (24 hours vs 7.40 hours). The time to start weaning was longer in the prospective group, and the same occurred with the stay in the unit. The rate of reintubations was lower with the application of a multidisciplinary protocol.

The differences found in the variables analyzed were related to the application of the multidisciplinary protocol. In the retrospective group, the most used ventilatory mode was Synchronized Intermittent Mandatory Ventilation or SIMV (70%) versus C/A (100%) in the prospective group as shown in Table 2. Ventilation times with O2 in T also varied between the two groups, with a predominance of times greater than 2 hours in the case of the retrospective cohort (Table 2).

**Table 2:** Ventilation modes and O 2 in T*. Elche, CV, Spain, 2015

Ventilatory mode	Retrospective Cohort (n=16)			Prospective Cohort (n=9)		
	%	fi	ni	%	fi	ni
SIMV^†^	68.75	11	0.68	0	0	0
C/A^‡^	31.25	5	0.31	100	9	1
O2 time in T* > 2 hours	75	12	0.75	0	0	0
Nurse participation: yes	0	0	0	100	9	1

* O2 in T = Oxygen with part in T;

† SIMV = Synchronized Intermittent Mandatory Ventilation;

‡ C/A = Controlled assisted

When analyzing which variables of those studied were the most closely related to IMV time, it was observed how in the retrospective group the increase in IMV time was associated with hospital stay (p<0.01), weaning duration time (p=0.019) and the time it takes to initiate the weaning process since the patient’s admission (p=0.013) as shown in Table 3. In the prospective group, the factors associated with increased mechanical ventilation time were the time between the start of the weaning (p=0.006) and hospital stay (p=0.003).

**Table 3:** Factors associated with invasive mechanical ventilation time. Elche, CV, Spain, 2015

Variables	Retrospective data (n=16) p value	Prospective data (n=9) p value
Sex vs MVT*	0.660	0.796
Age vs MVT*	0.780	0.271
Type of surgery vs MVT*	0.35	0.441
CCI^†^ vs MVT*	0.234	0.44
ASA^‡^ vs MVT*	0.972	1.00
Staying in days vs MVT*	<0.01	0.003
Weaning in hours vs MVT*	0.019	0.898
Time until weaning starts since entry vs MVT*	0.013	0.006
Reintubations: no(%) vs TVM*	0.323	0.04

* MVT = Mechanical ventilation time;

† CCI = Charlson Comorbidity Index;

‡ ASA = American Society of Anesthesiologists

## Discussion

The study attempts to reflect a reality in the practice of care in critical care units. In this case, the protocol applied is adapted to the characteristics of the surgical patient and to the need for a multidisciplinary approach considering the collaboration between doctor and nurse as something very relevant. Given the results obtained, the protocol could be applied in care units with similar characteristics, thus facilitating the integration of the nurse in a more active and participatory way in the process of releasing the IMV, something that has been proven to be positive in previous studies already carried out^(^ 9 ^-^ 10 ^,^ 31 ^)^.

According to Cochrane^(^ 9 ^)^, with the implementation of weaning protocols, the hospital stay in the critical care unit is reduced by 11%. Gupta et al.^(^ 13 ^)^ applied protocols to patients with simple and difficult weaning; obtaining an average of stay in unit between 12 and 26 days. In our case, the time of stay in the unit was shorter in the group to which the protocol was not applied, probably due to the associated comorbidities to the patient, generating a prolonged hospital stay in spite of having solved the main problem that originated the need for mechanical ventilation. It is important to point out that the increase in hospital stay is not related to the increase in mechanical ventilation time, nor to an increase in weaning time, so the application of the protocol is interesting in terms of reducing the complications associated with mechanical ventilation, and the causes of the increase in hospital stay may be related to the small sample used.

The delay time to start the release process from the intubation of the patient also varies when comparing both groups, being a longer time in the case of the application of the protocol. This difference is mainly because when the weaning protocol is applied, patients are subjected to a very rigid and complete evaluation of criteria that must be fully met in order to be candidates for extubation. These criteria are not present in the retrospective group, so that not all of them can be evaluated or patients can be catalogued as candidates according to individual criteria and the weaning process begins early. In previous studies consulted, the time taken to start the weaning process is not analyzed, so a comparison and a discussion are not possible.

A weaning process performed later in the prospective group has not influenced the time to achieve decreases in weaning time and overall IMV time, since although weaning starts later with the application of the protocol, once started, the time spent is less. This difference between the groups is probably due to the protocol with established times and the inclusion of the nurse within the process. The including of the nurse supposes the presence of a greater agility and continuity in the evaluation due to the multidisciplinary approach, since once the patient fulfills the criteria for the beginning of weaning evaluated by the doctor is verified, it is the nurse who initiates the reductions of SP and verifies the stability of the patient to the changes made with clearly established intolerance criteria. Previous studies had already reported the importance of the nurse to assess the patient’s ability and likelihood of success with the weaning process^(^ 9 ^-^ 10 ^,^ 31 ^)^.

In the retrospective cohort, mechanical ventilation time and weaning duration time are prolonged, probably because the responsibility for the management of the patient rests exclusively on the doctor, so the doctor can be more conservative, performing revaluations more spaced and at individual criteria. In previous studies ^(^ 3 ^,^ 9 ^-^ 10 ^,^ 22 ^,^ 26 ^-^ 27 ^)^, it is shown how the application of protocols influences in the reduction of the time that the process lasts, diminishing the time of weaning in a 70%^(^ 9 ^)^ and the total time of mechanical ventilation in a 26%^(^ 9 ^)^. In our case, we reduced weaning time by almost 17 hours, a number very similar to that obtained in previous studies^(^ 10 ^)^ and the total mechanical ventilation time by approximately 29 hours, a number that also approximates that obtained in previous publications^(^ 3 ^)^.

Although our results are in line with the bibliography previously presented, there are distinctions that make a difference with respect to previous studies, such as the presence of a joint evaluation, not only based on clinical parameters, but also on general conditions and foreseeable clinical evolution such as the need for close surgery or procedures in which it is preferable to have the airway insured. As an advantage and novelty, we point out the involvement of the nurse when making decisions and the great level of detail regarding the steps to follow when weaning the patient, trying to detect previously the patients with the maximum guarantees of carrying out the process in a safe and uncomplicated way. Thus avoiding making changes too quickly or too slowly without well-defined tolerance criteria that could lead to respiratory failure.

However, it is evident that our study has limitations because of the sample size and the comparison with a retrospective cohort. The limited sample size is due to our unit has a limited volume of patients and with a high turnover. This situation leads us to have short stays that do not meet the criterion of more than 24 hours of IMV compared to other patients with high probabilities of having a tracheostomy, as in the case of neurosurgical patients with important sequelae who do not meet the criterion of GCS superior than 9 to initiate the protocol.

In the retrospective group, after theoretically developing the weaning protocol and based on the bibliography regarding the improvement of quality and results after the application of these protocols, it did not seem ethical to us to propose a control group that would not be benefited by this improvement.

Due to the great advance represented by the inclusion of this work in our unit and the improvements obtained in the time of mechanical ventilation, it was decided to continue with the study in order to obtain a larger and more representative sample of patients that will allow in the future obtaining results that are more conclusive.

## Conclusion

With the implementation of a disconnection protocol carried out in a multidisciplinary way and giving a leading role to the nurse, it is possible to reduce the ventilatory time, the time spent on weaning and the numbers of reintubations, all without negative repercussions on the patient’s health.

The fact that it includes a joint evaluation of doctor and nurse with standardized disconnection criteria that the patient must comply entirely, favors a release process starting later. Although this fact may seem to be a disadvantage, it adds security to the protocol and allows for the inclusion of patients who are really candidates, thus making the process more agile and spending less time in the weaning process, which in turn reduces the total mechanical ventilation time.

However, it must be considered that despite the benefits obtained, the protocol not reduce the time spent in the critics unit. This may be due to the associated comorbidities of the patient that may influence the recovery process despite having managed to solve the respiratory problem and achieving a successful extubation.

Therefore, the implementation of this type of protocol in Spain, where the figure of the respiratory physiotherapist does not exist, should be considered as an effective method that provides improvements and benefits, and could become an important advance in terms of reducing the complications associated with mechanical ventilation and improving the management of these patients.
